# Supplementary material for: Self-Healable, Strong, and Tough Polyurethane Elastomer Enabled by Carbamate-Containing Chain Extenders Derived from Ethyl Carbonate
Source: Polymers (Basel). 2022 Apr 20;14(9):1673. doi: 10.3390/polym14091673 (PMC9101531; doi:10.3390/polym14091673)
Supplement: Supplementary file 1 [file polymers-14-01673-s001.zip › polymers-1663019-supplementary.pdf]

Supplementary Information

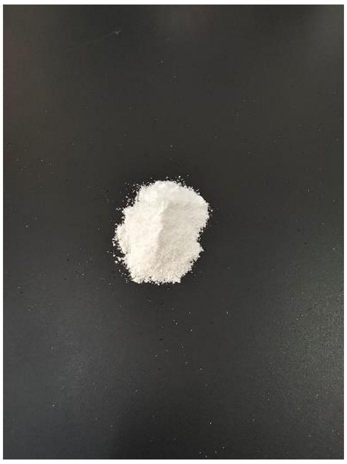

Figure S1. BDM powder.

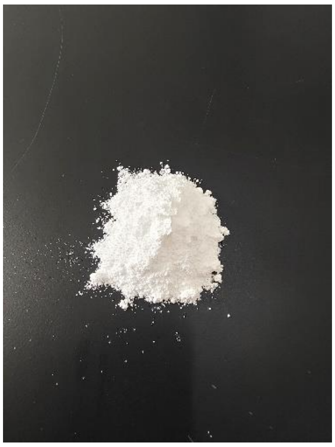

Figure S2. BDH powder.

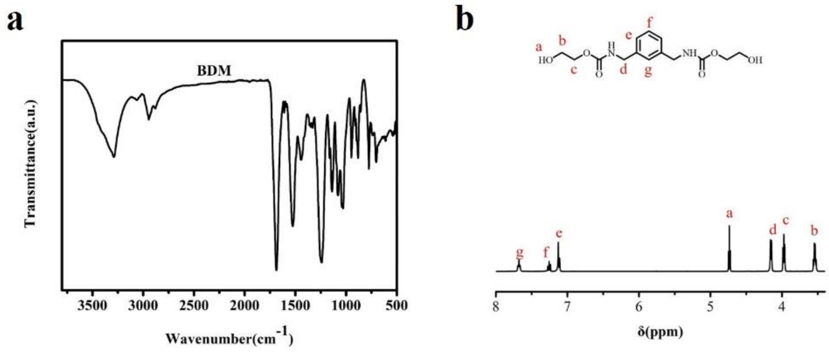

Figure S3. (a) FTIR spectra of BDH; (b) ^{1}HNMR spectrum of BDH.

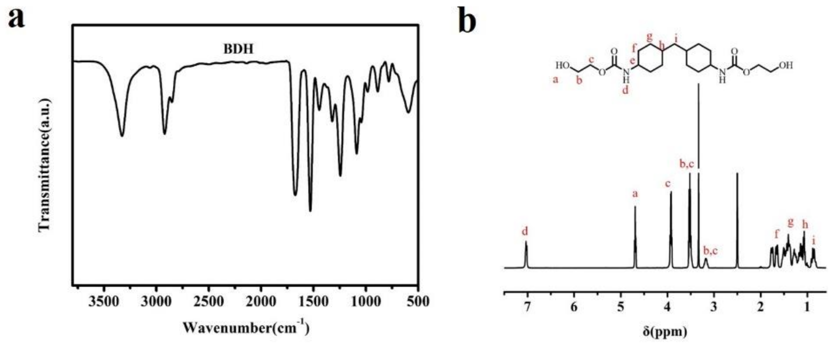

Figure S4. (a) FTIR spectra of BDH; (b) ^{1}HNMR spectrum of BDH.

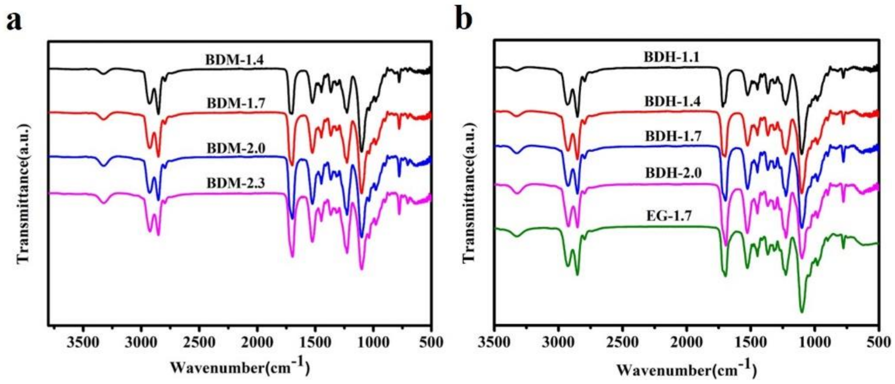

Figure S5. (a) FTIR spectra of BDM-based elastomers; (b) FTIR spectra of BDH-based and EG-1.7 elastomers.

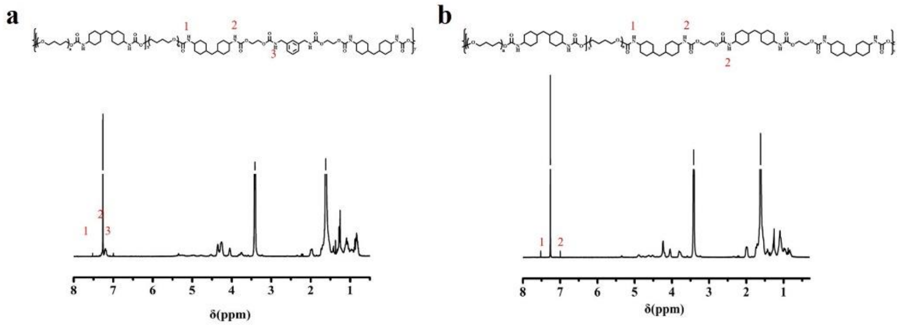

Figure S6. (a) ^{1}HNMR spectrum of BDM-1.7 elastomer, chemical shift for peak 1, 2 and 3 is 7.51, 7.20 and 7.00 ppm, respectively; (b) ^{1}HNMR spectrum of BDH-1.7 elastomer, chemical shift for peak 1 and 2 is 7.55, 7.01 ppm, respectively.

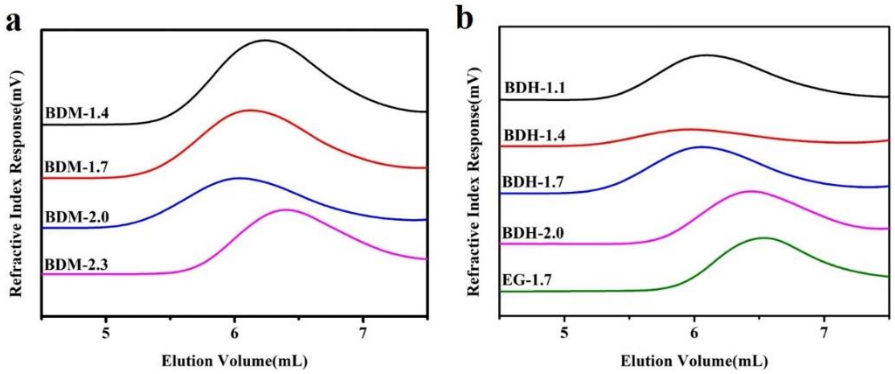

Figure S7. (a) GPC profiles for BDM-based elastomers; (b) GPC profiles for BDH-based and EG-1.7 elastomers.

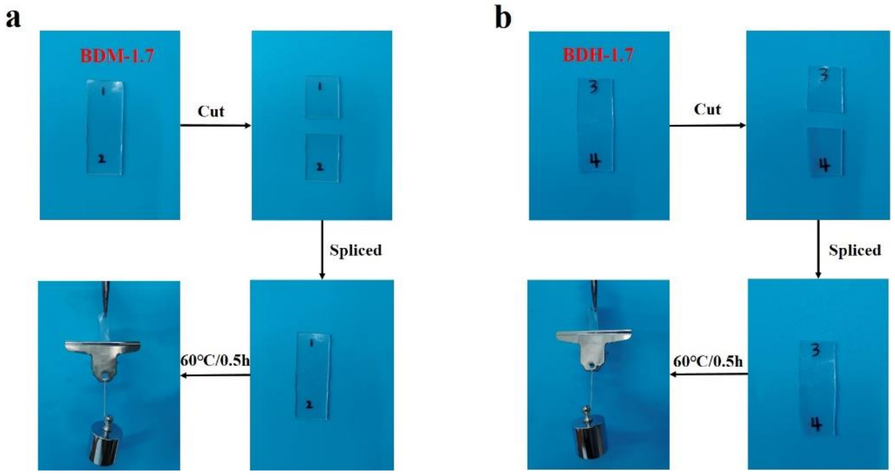

Figure S8. (a)and (b) The photos show that BDM-1.7 and BDH-1.7 elastomer tapes can be cut and processed at 60 °C for 0.5 h after splicing, which can increase the load of 500 g.

Table S1. Characteristic peak assignments of BDM.

Assignments	Wavenumber (cm^{-1})
ν (O-H)	3450,3411
ν (N-H)	3289
$\nu_{s\text{-}trans}$ (N-H)	3063
ν_{as} (CH_2)	2928
ν_a (CH_2)	2852
ν (C=O)	1687
m-benzene ring	1603,1593,1493, 777,704
δ (N-H)	1525
ν (C-O-C)	1138
ν (C-OH)	1044

Table S2. Characteristic peak assignments of BDH.

Assignments	Wavenumber (cm^{-1})
ν (N-H)	3324
$\nu_{s\text{-}trans}$ (N-H)	3060
ν_a (CH_2)	2921
ν_s (CH_2)	2855
ν (C=O)	1689
δ (N-H)	1528
ν (C-O-C)	1133
ν (C-OH)	1041

Table S3. Ingredients of BDM-based elastomers.

Sample	R value	Molar feed ratio [a]	Hard segments (wt%)	
			Short	Long
BDM-1.4	1.4	1 : 1.4 : 0.4	10.5	22.5
BDM-1.7	1.7	1 : 1.7 : 0.7	4.7	35.2
BDM-2.0	2.0	1 : 2.0 : 1.0	0	45.6
BDM-2.3	2.3	1 : 2.3 : 1.3	0	50.2

[a] PTMEG : HMDI : BDM.

Table S4. Ingredients of BDH-based elastomers.

Sample	R value	Molar feed ratio [a]	Hard segments (wt%)	
			Short	Long
BDH-1.1	1.1	1 : 1.1 : 0.1	10.9	13.8
BDH-1.4	1.4	1 : 1.4 : 0.4	10.4	23.9
BDH-1.7	1.7	1 : 1.7 : 0.7	4.6	37.2
BDH-2.0	2.0	1 : 2.0 : 1.0	0	53.7
EG-1.7	1.7	1 : 1.7 : 0.7	5.3	27.6

[a] PTMEG : HMDI : BDH.

Table S5. Characteristic peak assignments of BDM-based elastomers.

Assignments	Wavenumber (cm^{-1})
free ν (N-H)	3454
H-bonded ν (N-H)	3323
ν_a (CH_2)	2928
ν_s(CH_2)	2852
ν_s(CH_2)	2796
Free ν(C=O) amide I of urethane	1717
H-bonded ν(C=O) amide I of urethane	1697
m-benzene ring	1609, 777,704
δ (N-H)	1525
ν (C-O-C)	1100

Table S6. Characteristic peak assignments of BDH-based elastomers.

Assignments	Wavenumber (cm^{-1})
free ν(N-H)	3450
H-bonded ν(N-H)	3321
ν_a (CH_2)	2921
ν_s (CH_2)	2851
ν_s (CH_2)	2796
free ν (C=O) amide I of urethane	1721
H-bonded ν (C=O) amide I of urethane	1693
δ (N-H)	1526
ν (C-O-C)	1103

Table S7. Information of molecular weight of BDM-based elastomers.

Sample	M_n(g/mol)	M_w(g/mol)	PDI [a]
BDM-1.4	1.312×10^5	2.722×10^5	2.08
BDM-1.7	1.599×10^5	3.557×10^5	2.23
BDM-2.0	1.883×10^5	4.436×10^5	2.36
BDM-2.3	9.524×10^4	1.822×10^5	1.91

[a] polydispersity index.

Table S8. Information of molecular weight of BDH-based and EG-1.7 elastomers.

Sample	M_n(g/mol)	M_w(g/mol)	PDI [a]
BDH-1.1	1.585×10^5	3.465×10^5	2.19
BDH-1.4	2.446×10^5	4.913×10^5	2.00
BDH-1.7	1.940×10^5	4.086×10^5	2.10
BDH-2.0	9.635×10^4	1.697×10^5	1.76
EG-1.7	6.996×10^4	1.409×10^5	2.01

[a] polydispersity index.

Table S9. Mechanical properties of BDM-based elastomers obtained from tensile tests.

Sample	Tensile strength (MPa)	Elongation at break (%)	Toughness (MJ/m^3)
BDM-1.4	0.61 ± 0.03	805 ± 52	3.73 ± 0.19
BDM-1.7	20.6 ± 1.3	1667 ±56	83.5 ± 2.0
BDM-2.0	36.3 ± 1.3	1230 ± 61	122.3 ± 4.0
BDM-2.3	43.9 ± 2.4	1144 ± 40	135.8 ± 6.8

Table S10. Mechanical properties of BDH-based and EG-1.7 elastomers obtained from tensile tests.

Sample	Tensile strength (MPa)	Elongation at break (%)	Toughness (MJ/m^3)
BDH-1.1	0.19 ± 0.01	608± 93	0.58 ± 0.29
BDH-1.4	12.9 ± 0.7	1740 ± 25	57.7 ± 1.1
BDH-1.7	37.1 ± 1.7	1128 ± 53	118.8 ± 5.1
BDH-2.0	44.0 ± 3.3	1079 ± 20	197.9 ± 7.9
EG-1.7	0.13 ± 0.02	491 ± 30	0.43 ± 0.1

Table S11. The Self-healing efficiencies of BDM-1.7 elastomers.

	Sample	25 °C/24h	40 °C/12h	60 °C/24h
Tensile strength S.E. (%)	BDM-1.7	24.7	60.4	99.8
Elongation at break S.E. (%)	BDM-1.7	64.9	90.0	100.0
Toughness S.E. (%)	BDM-1.7	30.2	67.7	98.8

Table S12. The Self-healing efficiencies of BDH-1.7 elastomers.

	Sample	25 °C/24h	40 °C/12h	60 °C/24h	60 °C/4h	60 °C/8h	60 °C/12h
Tensile strength S.E. (%)	BDH-1.7	19.2	64.0	95.2	50.7	61.6	77.0
Elongation at break S.E. (%)	BDH-1.7	52.2	87.1	99.4	81.5	87.3	93.2
Toughness S.E. (%)	BDH-1.7	16.7	67.8	95.6	51.6	59.4	79.7
